# Improving signal-to-noise ratio in transcranial magnetic resonance guided focused ultrasound

**DOI:** 10.1186/2050-5736-3-S1-O36

**Published:** 2015-06-30

**Authors:** Rock Hadley, Dennis Parker, Emilee Minalga

**Affiliations:** 1 University of Utah, UT, United States

## Background/introduction

Signal-to-Noise Ratio (SNR) can be increased in Magnetic Resonance Imaging (MRI) using Radio Frequency (RF) coils. Of all the options for increasing SNR, coils provide the greatest gains for the dollars spent. Coils for 1.5 and 3 Tesla MRI systems consist of conductive loops that are tuned to resonate at the fundamental frequency associated with the field strength of the MRI scanner. These coils are the transducers between the MR signal and the system electronics and are sensitive to the magnetic fields of the MR signal via Faraday’s Law of Maxwell’s equations. Sensitivity of a coil to the signal in the imaging sample depends primarily on the geometry and position of the loop with respect to the sample. To achieve the highest SNR requires the coil to be in close proximity to the sample of interest, small enough that it is not sensitive to regions outside the region of interest and large enough to pick up the signal at the depth of interest. Other factors such as dielectric or conductive material loading of the coils can significantly affect the tuning and function of a coil. Custom coils designed for a specific application typically provide much higher SNR than commercial coils that are designed for general purpose imaging of a broad range body habitus. Coils that provide even small gains in SNR provide significant imaging improvement. For example, a specific-purpose (SP) coil that can provide a 40% SNR improvement over a general-purpose (GP) coil, can achieve the same image quality as the GP coil in half the imaging time. Similarly, an SP coil that can provide a factor of 2 improvement in SNR over a GP coil can achieve the same image quality 4 times faster than the GP coil. SNR can be used to improve image quality, temporal and spatial resolution, and enable or improve imaging functionality such as temperature measurement accuracy, Diffusion Tensor Imaging (DTI), and MR Acoustic Radiation Force Imaging (MR-ARFI).

## Methods

TcMRgFUS systems present many obstacles to achieving maximum SNR. Specifically, the large US transducer array with its associated housing and cables, the water bath required for US transmission, the membrane that contains the water and its position on the head (the head is partly in and partly out of the water), the stereotactic head frame, and the requirement that RF coils cannot interfere with the US beam make RF coil design for this application very difficult at best. In addition to signal reception, signal transmission can be difficult with the US housing shielding the sample from the transmit coil, resulting in an inhomogeneous or reduced flip angle across the volume of the sample. The large water bath can cause standing wave effects in the transmit RF field and moving water can cause motion artifacts in the images. Because of all these imaging obstacles, it’s easiest to implement the use of system body RF coils for signal transmission and reception. Because of their size and distance from the imaging volume, they are inherently more forgiving to variable loads like the TcMRgFUS system. However, they also provide relatively low SNR, and are clearly not sufficient for high-end imaging applications. Some research groups working on TcMRgFUS are using coils that have been developed specifically for that application. Some of the coils currently in use consist of arrays of coils that are wrapped around or integrated into the housing of the TcMRgFUS system. Others use arrays of coils that fit close to the head. The close fitting coils can be inside the water bath and outside the water bath. One institution is using coils that use conductors that cross the water bath membrane where part of the loop is in the water and a part of the same loop is outside the water. Watkins et. al. at Stanford have developed a 2-part birdcage-like Transmit/Receive coil where one half is in the water bath and the other half is outside the water bath. The two halves are inductively coupled to generate a homogenous field throughout the volume of the brain. This coil design also eliminates the need to use the body coil for transmission and therefore avoids the shielding effects that can be associated with use of the body coil transmission through the US transducer. This coil does not, however, enable parallel imaging techniques to be used as the multiple coil element arrays do.

## Results and conclusions

In each case where a dedicated RF coil is being used for TcMRgFUS, the SNR provided by the dedicated RF coil is significantly higher than the body coil of the MRI system by factors as much as 5x and more. Results of using the dedicated RF coils include improved anatomical imaging and improved temperature accuracy, which is important for patient safety, for improved assessment of thermal dose and efficacy, and for overall patient treatment planning.

It is difficult to make an accurate SNR comparison of the TcMRgFUS coils currently in use because the SNR measures are being done in different environments, across different MR platforms and with different transducer designs. As we gain experience with the RF coils that are currently being used and continue to push the MRg limits for FUS requirements in the human brain, coil designs with higher SNR will become more important. Achieving the ultimate SNR coil design will require the development of a TcMRgFUS system with the RF coils integrated into the design. Optimized RF coil design and implementation will prove to be a significant contribution for obtaining precise, accurate, and efficacious patient treatment planning for FUS treatments of brain disorders and disease.

